# Defined covalent assembly of protein molecules on graphene using a genetically encoded photochemical reaction handle†

**DOI:** 10.1039/c7ra11166e

**Published:** 2018-02-05

**Authors:** Athraa J. Zaki, Andrew M. Hartley, Samuel C. Reddington, Suzanne K. Thomas, Peter Watson, Anthony Hayes, Andy V. Moskalenko, Monica F. Craciun, J. Emyr Macdonald, D. Dafydd Jones, Martin Elliott

**Affiliations:** School of Physics and Astronomy, Cardiff University Cardiff CF24 3AA UK ElliottM@cardiff.ac.uk; School of Biosciences, Cardiff University CF10 3AX UK JonesDD@cardiff.ac.uk; Centre for Graphene Science, University of Exeter EX4 4QF UK

## Abstract

We have created modified protein variants by introducing a non-canonical amino acid *p*-azido-l-phenylalanine (azF) into defined positions for photochemically-induced covalent attachment to graphene. Attachment of GFP, TEM and cyt *b*_562_ proteins was verified through a combination of atomic force and scanning tunnelling microscopy, resistance measurements, Raman data and fluorescence measurements. This method can in principle be extended to any protein which can be engineered in this way without adversely affecting its structural stability.

## Abstract 2

We demonstrate a general method for photochemically-induced covalent attachment of proteins to graphene through the introduction of a non-canonical amino acid *p*-azido-l-phenylalanine into defined residue positions.

## Graphical abstract

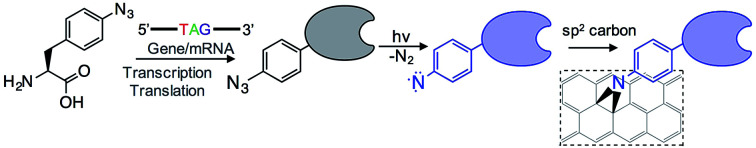

## 1 Introduction

The measurement and control of electron transport through single molecule junctions is an important step in the development of single molecule electronic components and chemical or biochemical sensors.^1–3^ More recently, due to their inherent molecular recognition properties, protein molecules have become a particular focus.^1^ Attachment of small organic molecules through thiol linkers^4^ to metal surfaces such as gold is now well established. We^5^ and others have shown how single protein molecules can by engineered with cysteine groups at precise location(s) in its 3D structure for defined thiol attachment to metal surfaces. Cysteine has the advantage compared to other commonly used reactive groups in proteins (*e.g.* amine and carboxyl groups) in that it is less numerous in proteins, so allowing a more defined coupling position. However, cysteine is still present in many proteins, and needs to undergo additional chemical modification to facilitate indirect interfacing with many electronically active non-metallic surfaces, such as graphene. This can result in the loss of intimate electronic coupling between protein and surface.

For practical applications it is crucial to attach molecules robustly and in controlled orientation on to technologically important electrodes such as graphene. An intimate attachment is required to optimise coupling of the molecular recognition function of a single protein molecule to the electronically active sp^2^ network of graphene. Homogeneous attachment is required to achieve consistent response of a multi-protein device.

Passive adsorption through hydrophobic interaction is commonly used to interface proteins and graphene^6^ but this gives weak, non-specific and non-homogeneous attachment. Covalent attachment^7^ is an attractive alternative but the graphene surface is largely inert chemically even for direct attachment of reactive thiol groups. Graphene thus needs to be chemically oxidised to introduce carboxyl, ketone and alcohol groups to facilitate covalent linkage. However, this introduces an extra processing step, can be difficult to control, and can potentially break both the σ and π bond networks that give graphene its important electronic characteristics and mechanical strength.^8,9^

Here we present an attractive alternative approach through the genetically encoded incorporation of useful reaction handles not present in natural proteins. Using a reprogrammed genetic code we introduce the non-proteinogenic, non-canonical amino acid (ncAA) *p*-azido-l-phenylalanine (azF) into a defined position in a protein. The protein can be directly covalently linked to graphene without the need to modify the base graphene surface by using the phenyl azide photochemistry inherent to azF.^7,10^ This approach is thus universal, in the sense that in principle any protein can be attached to graphene using this method as long as the azF non-canonical amino acid can be inserted into suitable positions on the protein surface without significantly affecting (in common with any other attachment methods) the protein structural and functional stability. Atomic force microscopy (AFM) imaging of a number of different proteins attached to graphene demonstrates the success of this method at the individual molecule level. This opens up the possibility of creating both graphene-based field effect transistors for a wide range of biological and chemical sensing applications, and bionanodevices more generally.

## 2 Background

The introduction of new physicochemical properties into proteins through genetically encoded unnatural amino acid incorporation, particularly phenyl azide chemistry, has previously been demonstrated by us.^10,11^ We summarise the technique here.

**Fig. 1:**
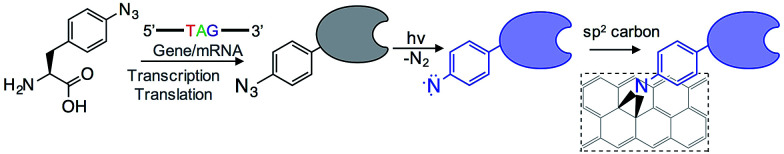
Outline of the coupling of proteins to graphene *via* the genetically encoded phenylazide photochemistry.

Using reprogrammed codon systems^12–14^ phenyl azide photochemistry can be incorporated at defined residues within a target protein^10,15^ by using the ncAA azF as indicated in Fig. 1. On irradiation with UV light the phenyl azide forms a reactive nitrene radical that effectively inserts between the C=C bonds in the sp^2^ network without introducing breaks within the C–C network (Fig. 1) and so has minimal effect on the conjugated π bond network.^6,7,16^ This photochemical coupling is particularly attractive as it is more controllable, temporally, spatially and energetically than chemical addition. Because the reactive handle is inherent to the protein there is no need to activate and thus disrupt the sp^2^ system prior to protein addition. Covalent coupling also provides a strong, intimate link between the two systems compared to approaches that require additional steps to attach intermediate moieties such as pyrene.^17–19^ It also allows far more control of position within the protein so as to optimise communication between the protein functional centre and the carbon surface; this is not possible with peptide handles that can only be attached to termini of a protein.^20,21^ Aromatic rings can also promote surface binding through π stacking so helping to place the nitrene close to the conjugated network.

To investigate the general utility of the method, three separate proteins with different structures and functions were chosen: TEM β-lactamase (BL),^22,23^ sfGFP,^24^ and cyt *b*_562_.

**Fig. 2:**
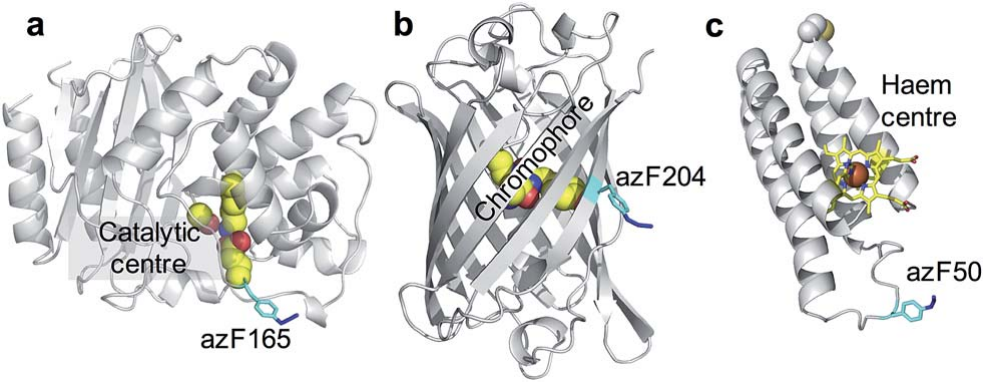
The position of azF residue in (a) TEM beta-lactamase (BL) (b) sfGFP and (c) cyt *b*_562_. The azF residue is shown in cyan and annotated with the residue incorporation position. The functional centres are coloured yellow and also annotated on each structure.

BL is a hydrolytic enzyme with α/β secondary structure (Fig. 2a) which is notorious in conferring antibiotic resistance through its breakdown of β-lactam antibiotics such as the penicillins.^25^ Coupling BL activity towards potential and existing antibiotics with graphene electronic output could provide a highly sensitive and miniaturised screening approach. The residue targeted, 165, lies close to the active site of BL and is known to tolerate azF incorporation and modification without significant affect on activity.^26^ The function of each variant described above is not affected by exposure to UV light.

SfGFP (Fig. 2b) is a predominantly β-sheet protein that belongs to the green fluorescent protein (GFP) class of auto fluorescent proteins.^27^ Amongst its many useful properties^28,29^ it also displays excited state proton transfer^30,31^ and conductance switching^32^ that can be coupled to, and thus modulated optically, the electronic properties of graphene. The residue targeted, 204, is known to tolerate azF incorporation without disruption to function and lies close to the chromophore maximising coupling to secondary components.^33^

Cyt *b*_562_ is a small α-helical bundle electron transfer protein (Fig. 2c) that binds the redox active cofactor haem 35–37. While it has potential as an active component in various nanoscience applications, it has recently emerged that cyt *b*_562_ has inherent transistor properties with conductance characteristics dependent on the redox state of the haem co-factor.^5,34^ Cyt *b*_562_ also tolerates azF incorporation (as shown by absorbance spectra) at various positions in its structure^35^ including the position targeted in this study, residue 50, which lies close to the haem cofactor (Fig. 2c) and is known to be an ideal coupling point to electrodes for single molecule analysis.^5,34,36,37^

## 3 Experimental methods

### 3.1 Protein mutagenesis

Two of the proteins of interest, TEM β-lactamase (BL)^26^ and super folder green fluorescent protein GFP (sfGFP)^10,33^ were mutated and produced as described previously. Briefly, the codon encoding residues 165 in BL and 204 in sfGFP were mutated to the amber stop codon (TAG). For cyt *b*_562_ gene encoding the SH-LA variant^5,36^ was cloned into the pBAD plasmid (termed pBAD-cyt *b*_562_). Amber stop codon (TAG) mutations were introduced by the Phusion whole plasmid site-directed mutagenesis PCR (NEB Biolab) using the pBAD-cyt *b*_562_ plasmid containing the wild type gene of interest as a template; oligonucleotide primers were designed to incorporate the TAG mutation at the desired position in the gene (SRc50TAGF; 5′-ACAGCCCGGAAATGAAAGATTTC3′; SRc50TAGR 5′-CCGGTGATTTCTATTCGAGCTTCG-3′).

### 3.2 Protein production

Two plasmids were required for incorporation of azF into proteins; pDULE,^38^ which carries the engineered tRNA and tyrosyl tRNA synthase for azF incorporation, and pBAD (Invitrogen) which carries the gene of interest with the desired TAG mutation. Both plasmids were used in equal concentration to transform *E. coli* TOP10 cell aliquots. Starter cultures of 5 mL LB broth (containing 25 μg mL^−1^ tetracycline and 100 μg mL^−1^ ampicillin) were inoculated with a single bacterial colony and incubated at 37 °C overnight. Larger expression cultures (10 mL to 1 L) of ZYM-5052 autoinduction medium 12 were inoculated with a 1/200 dilution from the saturated culture. An hour after inoculation, 1 mM azF was added and cultures were incubated at 37 °C for 24–30 hours. Cultures were grown in the dark to prevent photolysis of the azF by ambient light. Protein production was detected by SDS-PAGE and fluorescence spectroscopy for sfGFP. Protein were purified essentially as described previously: BL;^26^ sfGFP;^10,33^ cyt *b*_562_.^39,40^

### 3.3 Sample preparation and imaging

Binding to graphene was provided through covalent linking of the proteins to the surface stimulated by UV irradiation. Sample preparation was performed in a nitrogen-flushed glove box with UV-protective yellow film covering all the windows. It was important to perform experiments in low relative humidity of around 1%. The humidity is a proxy for oxygen content, which was not measured directly. UV exposure in the presence of oxygen results in oxidation of the graphene surface. Protein samples were deposited on monolayer graphene on copper foil substrates (Graphene Supermarket). Foil pieces (4 × 2 mm) were immersed in 10 mM phosphate buffered saline (PBS) containing protein at various concentrations in the range 1–10 nM, or 1 μM for Raman experiments, at room temperature. They were incubated for 10 minutes, either in the dark, or in the presence of UV irradiation with intensity of 8.7 Wm^−2^ provided by a UVM-57 hand-held UV lamp (302 nm, 6 watt) at a distance of 5 cm from the sample surface. Following incubation the samples were rinsed with deionised water, either by agitating the sample in a beaker for 1 minute or under flowing water (in this case, outside the glove box) for 5 minutes, and then dried with nitrogen gas. The samples were then attached to sample holders for subsequent atomic force microscopy (AFM) measurements.

All AFM imaging was performed using a Veeco Nanoscope IIa (Bruker) in tapping mode. Imaging was made for different regions to check the distribution of the protein molecules across the surface.

## 4 Results and discussion

It is known that^6,41,42^ proteins can adhere to graphene or other surfaces through largely hydrophobic interactions. Surface accessible aromatic groups on proteins can help facilitate and direct binding through π electron stacking. Therefore, to help observe individual molecules experiments were performed in which graphene was immersed with a low (1–10 nM) concentration of protein to generate sub monolayer coverage. From repeat experiments we observed distinct correlation between increase in protein concentration and in UV exposure time (from seconds to several minutes) with the final protein surface coverage measured with AFM. In this way the best conditions to observe individual molecules were found empirically. These conditions varied slightly for each protein, possibly due to differences in interaction and accessibility of the azide group with the graphene surface during incubation. The persistence of individual protein molecules could then be examined by AFM. Any protein on the surface after incubation in the dark would be expected to be smaller in number and weakly bound.

### 4.1 GFP204-azF

**Fig. 3:**
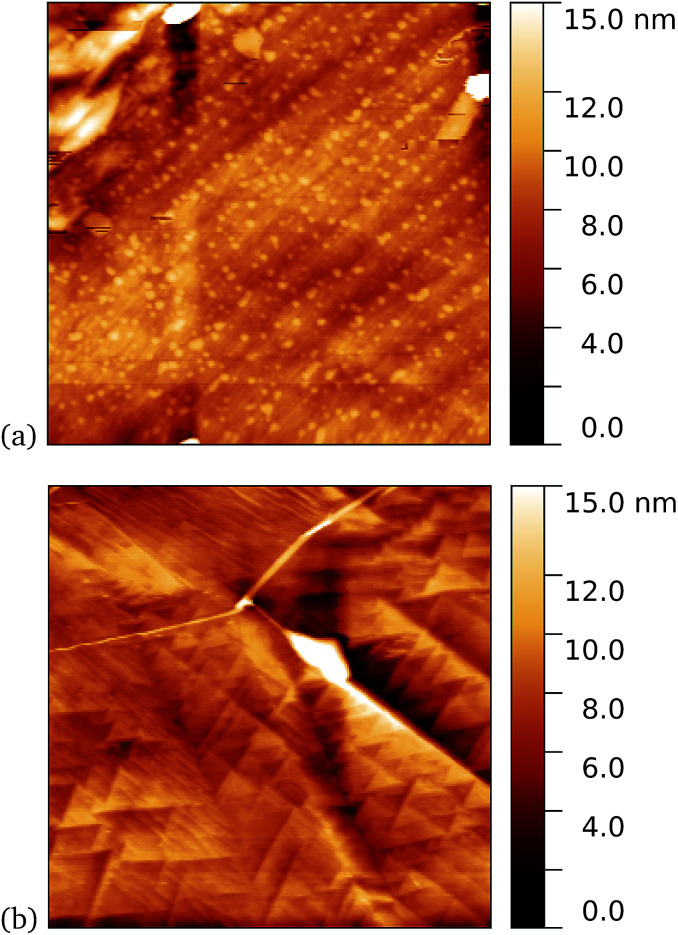
Results of incubation of 1 nM concentrations of GFP204-azF molecules with graphene for 10 minutes (a) with UV illumination and (b) in the dark, followed by a 1 minute rinse. Typical AFM images of 1 μm squares show a distinct difference in coverage between the dark- and UV-prepared sample. The proteins heights are around 3 nm.

As a first demonstration of the approach, sfGFP204-azF protein was deposited at 1 nM concentration while under UV exposure for 10 minutes, followed by a 1 minute rinse. AFM measurements, representative images of which are shown in Fig. 3, showed a distinct difference between samples incubated with UV exposure, Fig. 3a, and in the dark, Fig. 3b. Clear individual proteins of relatively uniform shape and apparent height up to 2–3 nm are observed in the first case. The expected height of the protein β-barrel lying parallel to the graphene is around 3 nm, but it is not unusual for apparent AFM heights of soft materials to be smaller than true heights.^43^

These results clearly suggest that while sfGFP204-azF shows little capacity bind to monolayer graphene in the dark, irradiation to photochemically generate the phenyl nitrene results in successful covalent interfacing of protein and graphene. Individual proteins can easily be imaged for this concentration and are fairly uniform in apparent size. Typical heights observed are spread around 3 nm (see ESI Fig. S1.†).

**Fig. 4:**
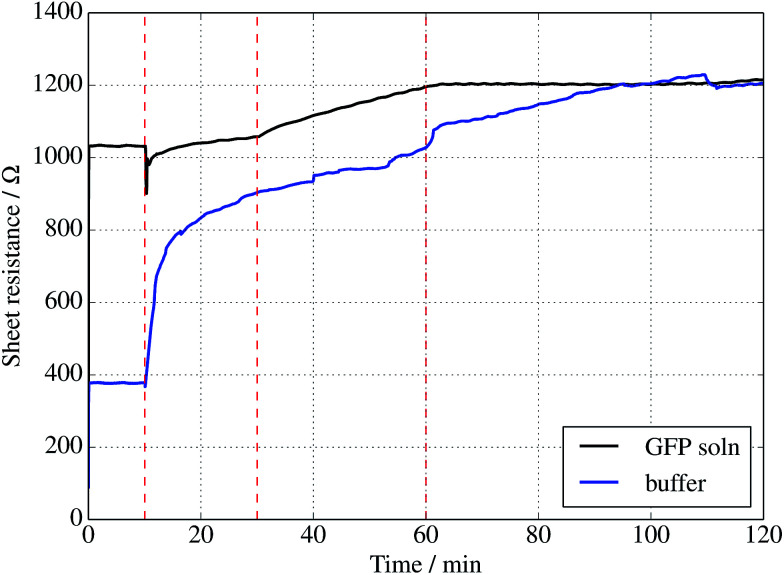
Sheet resistance of graphene measured in the presence of a 1 μM solution of sfGFP (black curve) solution or buffer solution alone (blue curve). In each case, a droplet was placed onto the centre of a 1 cm^2^ piece of graphene at 10 min, followed by illumination with a 305 nm UV diode between 30 and 60 min only.

Further evidence of binding can be provided through sheet resistance measurements. There are two effects to consider. Firstly, covalent functionalisation of graphene may result in a change in sheet resistance^6,44^ due to disruption of the sp^2^ lattice (which may open a gap at the Dirac point or a change the Fermi energy). Secondly, doping of the graphene due to charge transfer between the protein is generally possible. To examine resistance changes, a four terminal measurement of graphene on silicon was made in the presence of UV illumination while incubating proteins. This was done for a GFP solution as well as a buffer without GFP molecules present. Experiments were performed in a nitrogen-flushed glovebox to help reduce the surrounding oxygen concentration. As seen in Fig. 4, the graphene resistance increases with time in the presence of both buffer solution and GFP solution, suggesting a degree of oxidation. However, when the UV illumination is switched on at 30 minutes a distinct ‘kink’ (increase in the rate of change of resistance with time) is observed for the GFP-containing solution only, consistent with photoinduced binding between the azide and graphene. Another kink (rate decrease) is seen when the UV is switched off at 60 minutes. A simple charge transfer is possible, but the observation of a UV induced change strongly suggests that covalent binding is the cause.

**Fig. 5:**
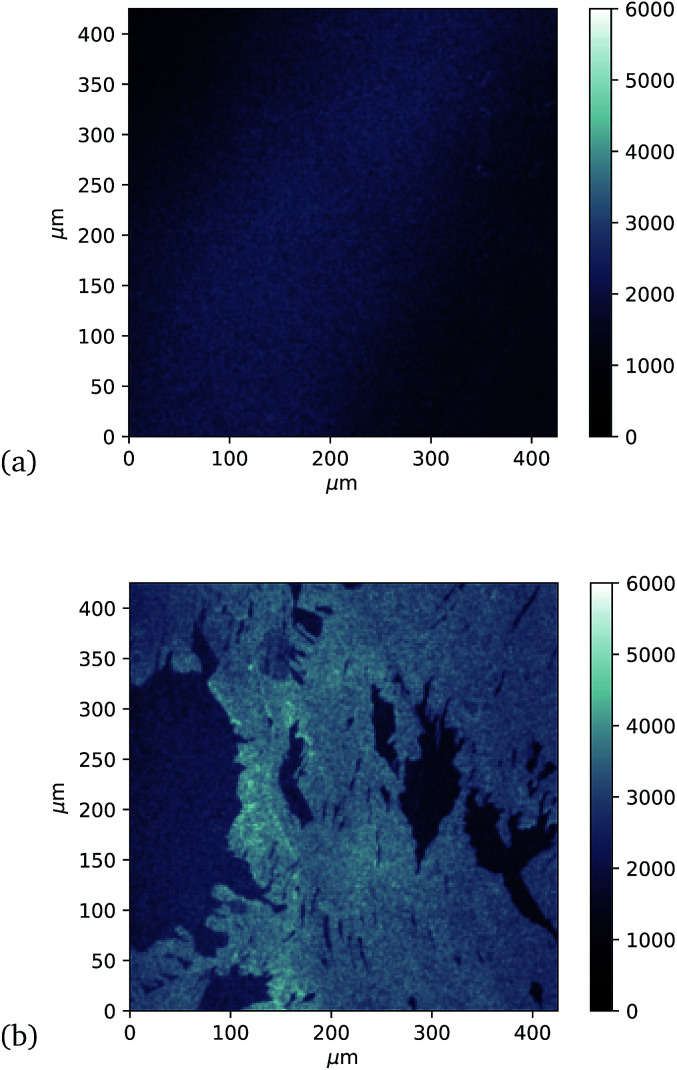
Approximately 400 μm square confocal microscopy images to compare fluorescence from separate single-layer graphene on silicon samples incubated with GFP204-azF molecules (10 nM) (a) in the dark and (b) with 10 minute UV exposure, each followed by thorough rinsing with deionised water for one minute. (The vertical intensity scale is arbitrary and the same for each sample.) Images taken using a Zeiss LSM880 confocal laser scanning microscope.

Finally, investigation of GFP attachment was verified using confocal microscopy to measure fluorescence of graphene after incubation with GFP204-azF molecules exposed to UV or in the dark, followed by rinsing. It is clear in Fig. 5 that proteins attach to the graphene only in the presence of UV treatment. (An area has been chosen in Fig. 5b to include partial graphene coverage to illustrate more clearly how proteins attach to the graphene. Other areas show fuller coverage.) These results were verified on other samples and areas examined and also demonstrate that the GFP has maintained sufficient functionality to fluoresce.

To demonstrate the wider applicability of the approach in terms of the proteins and their functional type, TEM and cyt *b*_562_ were examined in a similar way.

### 4.2 TEM105-azF and TEM165-azF

**Fig. 6:**
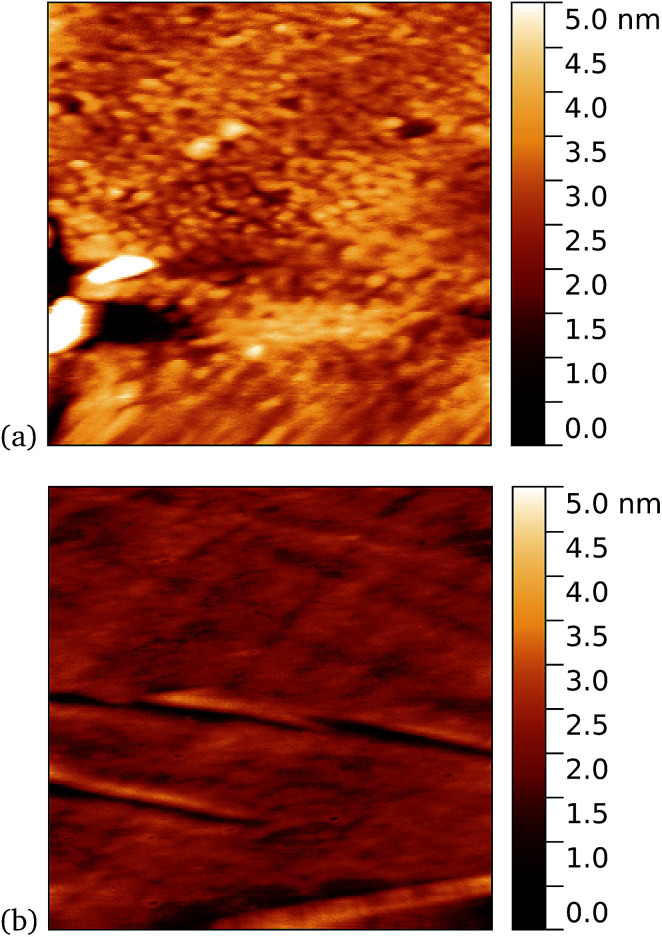
Results of incubation of 10 nM concentrations of TEM105-azF molecules with graphene for 10 minutes (a) with UV illumination, 0.6 μm AFM image and (b) in the dark, followed by a 1 minute rinse, 1 μm AFM image. Regions of high coverage are found for UV but not for the dark.

**Fig. 7:**
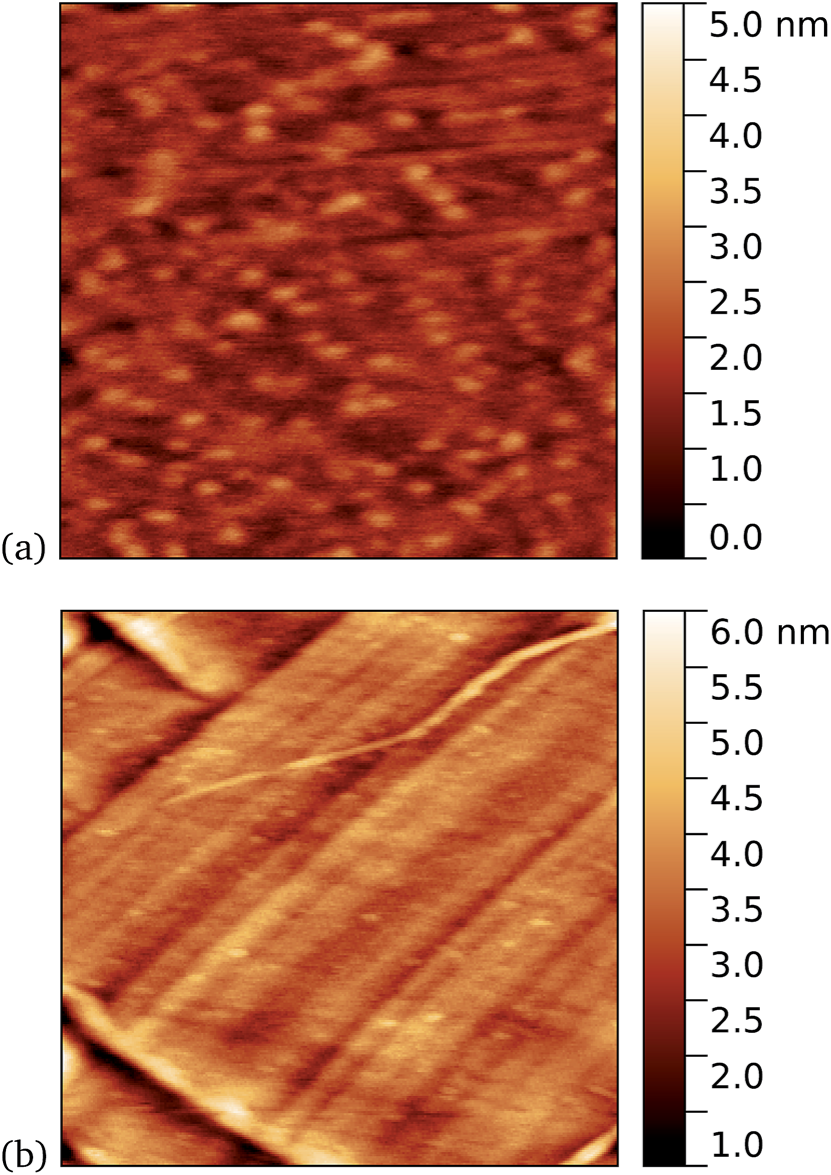
Results of incubation of 5 nM concentrations of TEM165-azF molecules with graphene for 10 minutes (a) with UV illumination, 0.6 μm AFM image and (b) in the dark, followed by a 1 minute rinse, 1 μm AFM image. Regions of high coverage are found for UV. A few proteins are also seen for the dark-prepared proteins.

Work by us^26^ and others^42^ has shown that TEM has a tendency to bind non-specifically and weakly to sp^2^ carbon systems. AFM results are presented for TEM105-azF and TEM165-azF in Fig. 6 and 7 respectively. Although a small protein density is visible in the dark in each case, UV treated samples shows a clear increase. There is some inhomogeneity of coverage across the graphene (probably related to the graphene itself: graphene on copper consists of mostly monolayers which have a varying degree of wrinkling) but the coverage is much greater with UV treated samples. Individual proteins were not easily distinguished for this high concentration, but again the typical molecular heights observed were 1–2 nm.

A very similar picture was found for TEM165-azF, as illustrated in Fig. 7. However in this case a few isolated single molecules are also found in the dark-prepared samples. With UV exposure more proteins are found on the surface, and they possibly form small aggregates, although in both cases the measured apparent heights are around 1–1.5 nm.

### 4.3 Cyt *b*_562_-azF

**Fig. 8:**
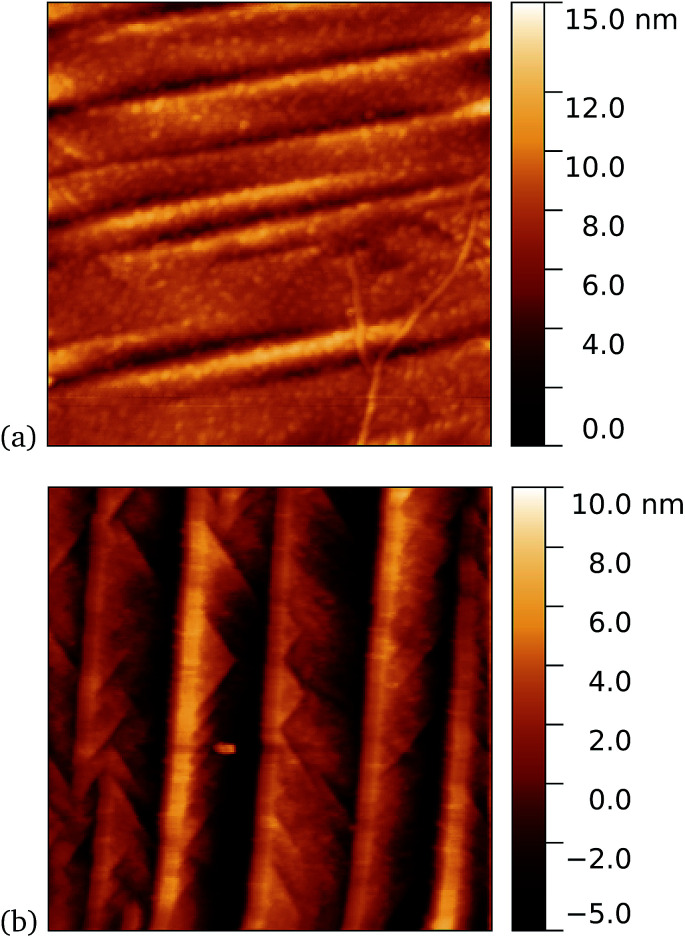
Results of incubation of 5 nM concentrations of Cyt *b*_562_-azF molecules with graphene for 10 minutes (a) with UV illumination and (b) in the dark, followed by a 5 minute rinse. AFM images of 1 μm square show regions of high coverage for UV but generally much less for the dark.

A similar trend was observed for cyt *b*_562_-azF50, with representative images shown in Fig. 8 for graphene incubated with 5 nM protein. Although, under these experimental conditions at least, cyt *b*_562_-azF proteins appear to adsorb non-specifically more easily onto graphene than the other proteins studied, UV treatment does increase the molecular coverage. Some inhomogeneity of coverage across the graphene was observed, but the coverage overall is greater with UV treated samples. Individual proteins can easily be imaged and are fairly uniform in apparent size. Typical heights observed are around 2 nm, which is a little smaller than the smallest 2.5 nm dimension of cyt *b*_562_, but considerably smaller than the expected dimension of 5 nm if the cyt *b*_562_ molecules are standing in the expected upright position.

**Fig. 9:**
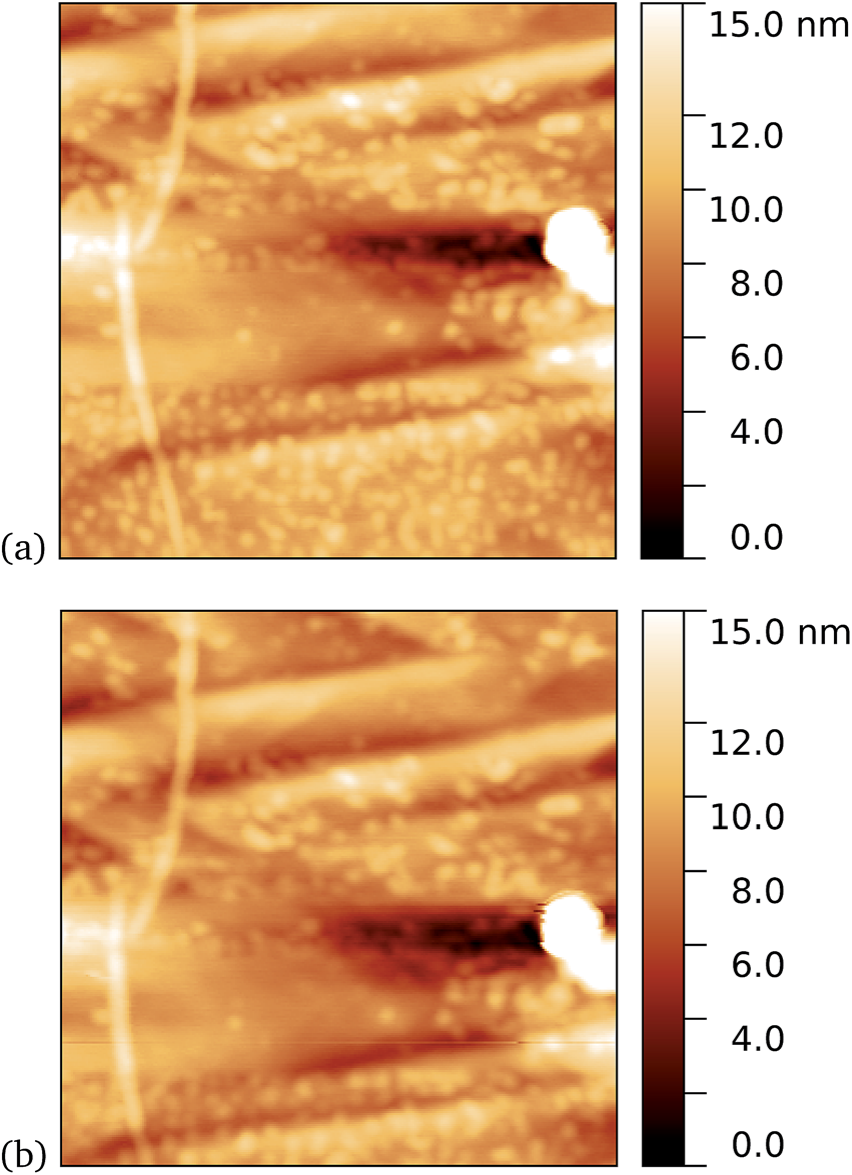
Results of incubation of 5 nM concentrations of cyt *b*_562_-azF molecules with graphene for 10 minutes with UV illumination, then rinsed for 15 minutes. AFM images of 0.59 μm squares show the proteins are robust to multiple scans. (a) 1st scan of area, (b) 10th scan. The heights of the proteins are around 2 nm.

We found a tendency for the AFM tip to become contaminated, as evidenced by consecutive scans becoming poorer, during scans of the dark prepared cyt *b*_562_-azF molecules. Wild-type TEM and cyt *b*_562_ molecules showed similar behaviour. In contrast, the UV-exposed samples were more robust to multiple imaging, as evidenced in Fig. 9. This is consistent with UV exposure resulting in covalent binding of the molecules, which are thus strongly attached to the graphene surface, rather than non-specifically bound molecules in the dark. Curiously, experiments with the wild-type molecules showed fewer molecules when a 10 minute UV incubation was used. The reason for this is not clear.

**Fig. 10:**
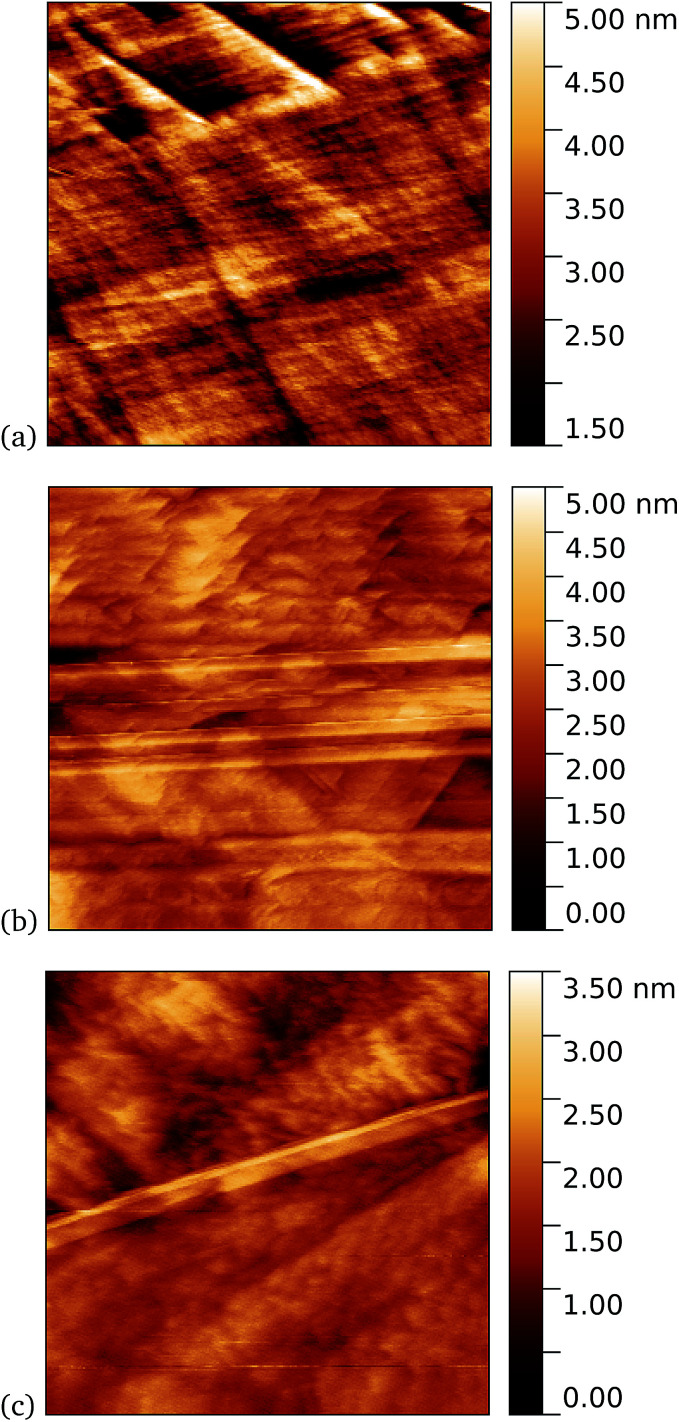
STM images. (a) A 1.1 μm square image of pristine graphene. After incubation of 10 nM concentration of cyt *b*_562_-azF molecules with graphene for 10 minutes (b) in dark, 0.9 μm square and (c) with UV illumination, 0.4 μm square.

Tapping mode AFM is a relatively non-invasive imaging mode for soft materials such as proteins. As such, it is not necessarily easy to remove non-specifically bound protein molecules that might adsorb to the graphene surface and which are not washed off during the rinsing procedure. It is therefore of interest to make scanning tunnelling microscopy (STM) imaging also, since this is a harsher technique which has the potential to sweep away weakly bound proteins. Imaging of cyt *b*_562_ on graphene might be expected to be possible since the molecules are intrinsically conducting in nature. Shown in Fig. 10 we observe a distinct difference between pristine graphene, samples prepared in the dark, and those prepared with UV illumination. Notably, for the 10 nM concentration used here, the protein coverage after UV illumination is very large and inhomogeneous, while it was possible to find only a few isolated proteins on the dark-prepared sample. This is strong evidence that UV treatment yields robust cyt *b*_562_ attachment.

**Fig. 11:**
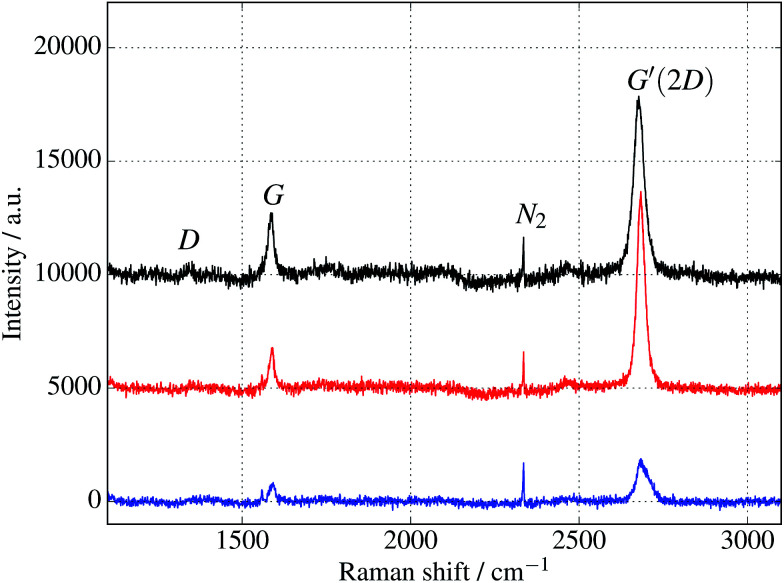
Raman spectra of cyt *b*_562_ taken with an excitation wavelength of 532 nm and spot size of 10 μm. (The background fluorescence signal has been removed and the curves are offset for clarity.) Top (black) curve is for untreated graphene. Middle (red) and bottom (blue) curves are for samples immersed in 1 μM protein solution for 10 minutes, in the dark and under UV illumination respectively. In addition to a general overall decrease of intensity, the ratios of the peaks change, as discussed in the text and shown in the ESI.†

Additional verification of covalent protein attachment was obtained by taking Raman spectra of the surfaces, shown in Fig. 11. There are three prominent features in each sample: the strong G′ (also denoted 2D) band centred around 2677 cm^−1^, the G band around 1585 cm^−1^, and a rather weak disorder-induced D band at around 1343 cm^−1^. The sharp peak around 2335 cm^−1^ is due to atmospheric molecular nitrogen.^45^ There is a consistent overall decrease in Raman intensity in the protein-treated samples, particularly after UV treatment. The G′/G ratio is strongly reduced for the UV-treated sample, as expected with doping of the graphene through linking to the azide group. Consistent with this, the G and G′ peaks are also upshifted (see ESI Fig. S6 and Table S2†).^46^ Finally, there appears a small sharp peak below the G band in the case of the UV exposed protein sample (and arguably weakly for the dark protein sample, but not pristine graphene) which can be attributed to the functionalisation of the graphene surface with proteins. Although covalent functionalisation of graphene might also be visible as an increase of the Raman D peak (due to disruption of the sp^2^ rings in graphene either by missing atoms or conversion of the sp^2^ bond into sp^3^ bond) the lack of observable D peak in the protein functionalised samples could also reflect the relatively small coverage of proteins; even a monolayer of proteins would only correspond to about 1% of bond disruption. Therefore the Raman measurements are only sensitive enough to detect doping effects due to charge transfer from covalently attached protein molecules.

## 5 Conclusions

We have shown experimentally that we can incorporate new chemistry into proteins to enable covalent linkage of protein molecules with graphene. We have demonstrated its feasibility with a variety of proteins with different functions and structures. The new chemistry can be introduced into a protein at a chosen residue position, allowing optimisation of the interface site and precise, defined linking with graphene. Importantly, this requires no pre-treatment of graphene for covalent functionalisation, because the linkage chemistry is inherent in the new non-canonical amino acid. This widely applicable method for insertion is light activated, so conditions are mild and biocompatible compared to some chemical treatments. This is supported by the retention of fluorescence of the GFP after attachment to graphene. Future work should include investigation of the broad structural and functional activity of the proteins after attachment, important to biocompatibility and general utility of the present methodology. Such work might include for example surface sensitive circular dichroism (CD) measurements which are sensitive to protein secondary structure. A combination of CD with other surface-specific techniques would help build a detailed picture of both ensemble averaged and single molecule information.

## Author contributions

AJZ performed experiments (sample preparation, STM/AFM imaging, resistance measurements) and contributed to Raman analysis. AMH designed and made proteins especially BL. SCR produced sfGFP protein and some cyt *b*_562_. AM provided work on glovebox protein preparation and STM operation. ST, AH and PW performed fluorescence measurements. MFC took the Raman data. JEM provided project supervision. DDJ wrote parts of the paper, provided data analysis and project supervision, and originated the binding concept. ME wrote parts of the paper, and provided Raman data analysis and project supervision.

## Conflicts of interest

There are no conflicts of interest to declare.

## Supplementary Material

RA-008-C7RA11166E-s001

## References

[cit1] Biju V. (2014). Chem. Soc. Rev..

[cit2] Heath J. R. (2009). Annu. Rev. Mater. Res..

[cit3] Song H., Reed M. A., Lee T. (2011). Adv. Mater..

[cit4] Xu B. (2003). Science.

[cit5] Della Pia E. A., Chi Q., Macdonald J. E., Ulstrup J., Jones D. D., Elliott M. (2012). Nanoscale.

[cit6] Georgakilas V., Otyepka M., Bourlinos A. B., Chandra V., Kim N., Kemp K. C., Hobza P., Zboril R., Kim K. S. (2012). Chem. Rev..

[cit7] Park J., Yan M. (2013). Acc. Chem. Res..

[cit8] Banhart F., Kotakoski J., Krasheninnikov A. V. (2011). ACS Nano.

[cit9] Aria A. I., Gani A. W., Gharib M. (2014). Appl. Surf. Sci..

[cit10] Reddington S., Watson P., Rizkallah P., Tippmann E., Jones D. D. (2013). Biochem. Soc. Trans..

[cit11] Reddington S. C., Rizkallah P. J., Watson P. D., Pearson R., Tippmann E. M., Jones D. D. (2013). Angew. Chem., Int. Ed..

[cit12] Wang L., Brock A., Herberich B., Schultz P. G. (2001). Science.

[cit13] Liu C. C., Schultz P. G. (2010). Annu. Rev. Biochem..

[cit14] Chin J. W. (2014). Annu. Rev. Biochem..

[cit15] Chin J. W., Santoro S. W., Martin A. B., King D. S., Wang L., Schultz P. G. (2002). J. Am. Chem. Soc..

[cit16] Han J., Gao C. (2010). Nano-Micro Lett..

[cit17] Chen R. J., Zhang Y., Wang D., Dai H. (2001). J. Am. Chem. Soc..

[cit18] Antonczak A. K., Simova Z., Tippmann E. M. (2009). J. Biol. Chem..

[cit19] Choi Y. (2012). Science.

[cit20] Cui Y., Kim S. N., Jones S. E., Wissler L. L., Naik R. R., McAlpine M. C. (2010). Nano Lett..

[cit21] Kim S. N., Kuang Z., Slocik J. M., Jones S. E., Cui Y., Farmer B. L., McAlpine M. C., Naik R. R. (2011). J. Am. Chem. Soc..

[cit22] Jelsch C., Mourey L., Masson J. M., Samama J. P. (1993). Proteins.

[cit23] Matagne A., Lamotte-Brasseur J., Frere J.-M. (1998). Biochem. J..

[cit24] Pédelacq J.-D., Cabantous S., Tran T., Terwilliger T. C., Waldo G. S. (2006). Nat. Biotechnol..

[cit25] Petrosino J., Cantu C., Palzkill T. (1998). Trends Microbiol..

[cit26] Hartley A. M., Zaki A. J., McGarrity A. R., Robert-Ansart C., Moskalenko A. V., Jones G. F., Craciun M. F., Russo S., Elliott M., Macdonald J. E., Jones D. D. (2015). Chem. Sci..

[cit27] Tsien R. Y. (1998). Annu. Rev. Biochem..

[cit28] Shaner N. C., Steinbach P. A., Tsien R. Y. (2005). Nat. Methods.

[cit29] Enterina J. R., Wu L., Campbell R. E. (2015). Curr. Opin. Chem. Biol..

[cit30] van Thor J. J. (2009). Chem. Soc. Rev..

[cit31] Remington S. J. (2011). Protein Sci..

[cit32] Korpany K. V., Langat P., Kim D. M., Edelman N., Cooper D. R., Nadeau J., Blum A. S. (2012). J. Am. Chem. Soc..

[cit33] Reddington S. C., Tippmann E. M., Jones D. D. (2012). Chem. Commun..

[cit34] Della Pia E. A., Chi Q., Jones D. D., Macdonald J. E., Ulstrup J., Elliott M. (2011). Nano Lett..

[cit35] Arpino J. A. J., Baldwin A. J., McGarrity A. R., Tippmann E. M., Jones D. D. (2015). PLoS One.

[cit36] Della Pia E. A., Macdonald J. E., Elliott M., Jones D. D. (2012). Small.

[cit37] Della Pia E. A., Elliott M., Jones D. D., Macdonald J. E. (2012). ACS Nano.

[cit38] Miyake-Stoner S. J., Refakis C. A., Hammill J. T., Lusic H., Hazen J. L., Deiters A., Mehl R. A. (2010). Biochemistry.

[cit39] Jones D. D., Barker P. D. (2004). ChemBioChem.

[cit40] Jones D. D., Barker P. D. (2005). Angew. Chem..

[cit41] Rajesh C., Majumder C., Mizuseki H., Kawazoe Y. (2009). J. Chem. Phys..

[cit42] Davis J. J., Green M. L., Allen H., Hill O., Leung Y. C., Sadler P. J., Sloan J., Xavier A. V., Chi Tsang S. (1998). Inorg. Chim. Acta.

[cit43] Yang C.-W., Hwang I.-S., Chen Y. F., Chang C. S., Tsai D. P. (2007). Nanotechnology.

[cit44] Sinitskii A., Dimiev A., Corley D. A., Fursina A. A., Kosynkin D. V., Tour J. M. (2010). ACS Nano.

[cit45] Hawaldar R., Merino P., Correia M. R., Bdikin I., Gracio J., Mendez J., Martin-Gago J. a., Singh M. K. (2012). Sci. Rep..

[cit46] Casiraghi C. (2009). Phys. Rev. B: Condens. Matter Mater. Phys..

