# Supplementary material for: Defined covalent assembly of protein molecules on graphene using a genetically encoded photochemical reaction handle
Source: RSC Adv. 2018 Feb 5;8(11):5768–75. doi: 10.1039/c7ra11166e (PMC9078156; doi:10.1039/c7ra11166e)
Supplement: RA-008-C7RA11166E-s001 [file RA-008-C7RA11166E-s001.pdf]

Electronic Supplementary Material (ESI) for RSC Advances.
This journal is © The Royal Society of Chemistry 2018

# Supporting information

## AFM analysis

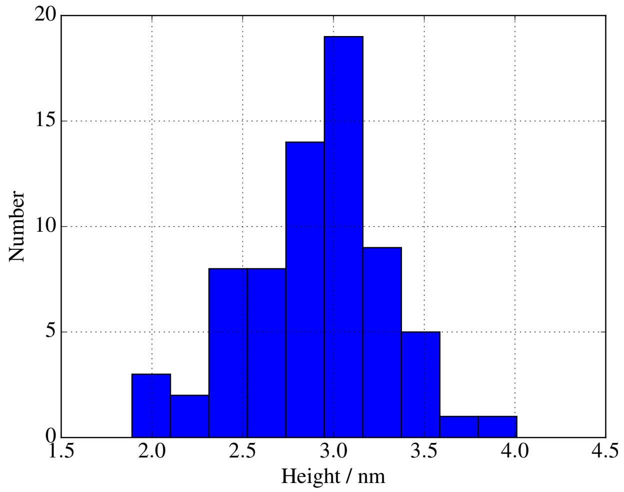


**Fig. S1** Height histogram obtained from GFP204-azF molecules shown in figure 3

## Raman analysis - GFP

Fits to portions of the raw Raman data of GFP are shown in figure S2 and summarised in table S1. The fitted parameters are shown in table S1.

| | G | | | G' | | |
|---|---|---|---|---|---|---|
| | Position / $cm^{-1}$ | Height (arb) | Width / $cm^{-1}$ | Position / $cm^{-1}$ | Height (arb) | Width / $cm^{-1}$ |
| **Pristine 1** | 1583.80 ± 0.33 | 1711.6 ± 39.9 | 12.64 ± 0.36 | 2666.71 ± 0.11 | 5369.7 ± 26.9 | 20.55 ± 0.13 |
| **Pristine 2** | 1586.50 ± 0.20 | 3073.0± 51.1 | 10.82 ± 0.22 | 2681.17 ± 0.13 | 5018.6 ± 30.4 | 19.41 ± 0.14 |
| **Pristine 3** | 1584.87 ± 0.28 | 2429.3 ± 55.9 | 10.69 ± 0.30 | 2677.36 ± 0.11 | 7064.3 ± 39.8 | 17.21 ± 0.12 |
| **Dark 1** | 1579.79 ± 0.52 | 848.4 ± 31.9 | 12.22 ± 0.56 | 2670.97 ± 0.19 | 2859.1 ± 21.8 | 22.82 ± 0.21 |
| **Dark 2** | 1580.85 ± 0.56 | 701.6 ± 27.5 | 12.81 ± 0.61 | 2670.77 ± 0.75 | 2360.5 ± 75.5 | 20.84 ± 0.81 |
| **Dark 4** | 1585.38 ± 0.84 | 661.6 ± 29.1 | 17.38 ± 0.97 | 2687.60 ± 0.40 | 1469.8 ± 18.5 | 28.95 ± 0.46 |
| **UV 1** | 1588.14 ± 0.55 | 748.0 ± 30.9 | 11.76 ± 0.59 | 2694.88 ± 0.36 | 1417.1 ± 21.3 | 21.97 ± 0.41 |
| **UV 2** | 1584.95 ± 0.25 | 3523.8 ± 72.0 | 10.81 ± 0.27 | 2675.71 ± 0.10 | 13609.4 ± 61.1 | 19.34 ± 0.11 |
| **UV 3** | 1585.82 ± 0.44 | 1104.9 ± 39.5 | 11.39 ± 0.49 | 2683.66 ± 0.24 | 2369.6.3 ± 21.2 | 24.607 ± 0.26 |

**Table S1** Comparison of main peak position, height and width for GFP Raman data The fits are shown in figure S2.

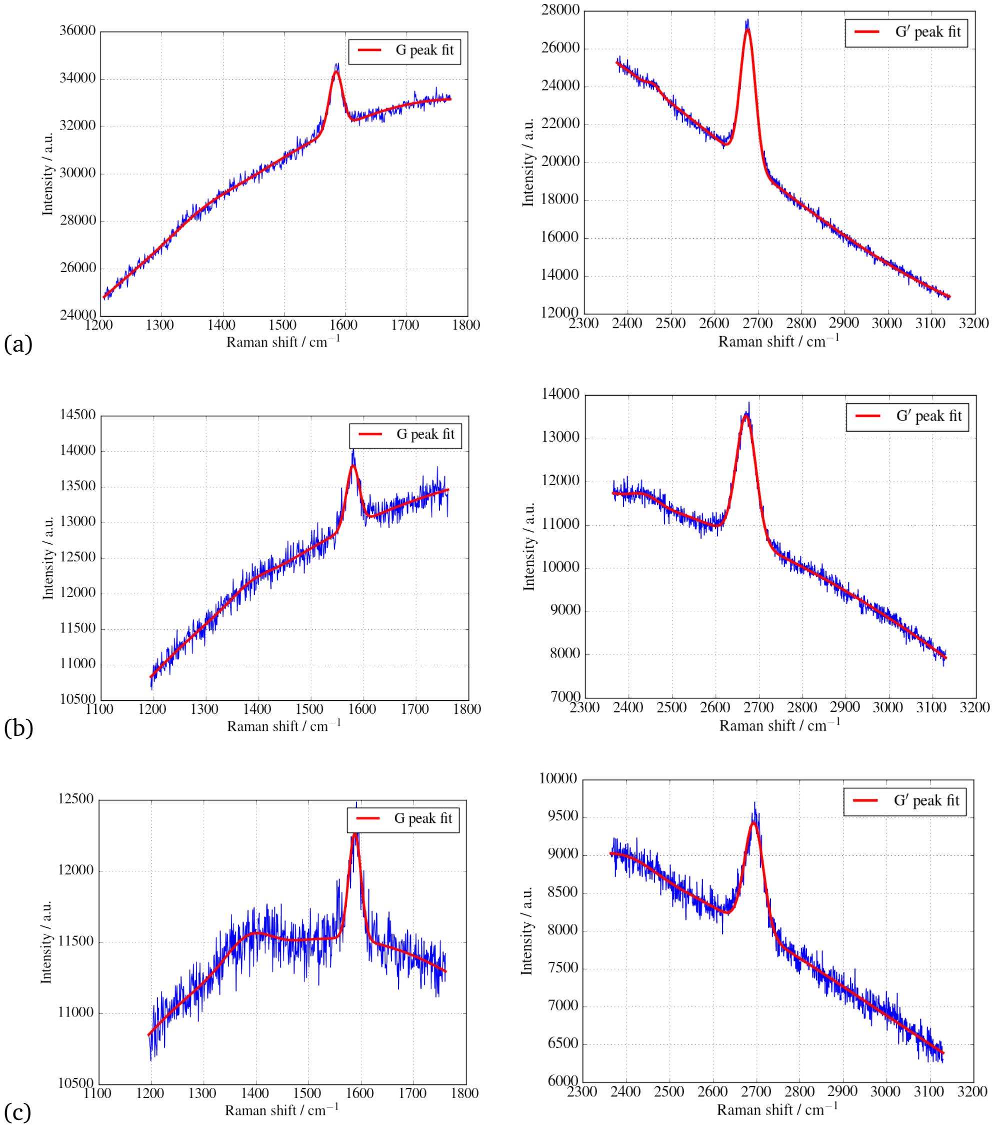


**Fig. S2** Selection of fits to raw Raman data (blue) are shown as red curves and comprise double Gaussian peaks with a cubic polynomial background. The left (or right) column shows data around the G' (or G) peaks. (a) pristine graphene (b) with proteins deposited in the dark (c) with proteins deposited with UV exposure. For clarity, the sharp feature below the G peak at around 1560 cm$^{-1}$ has not been fitted in these images.

## Raman analysis - cyt $b_{562}$

Fits to portions of the raw Raman data of cyt $b_{562}$ corresponding to figure 11 of the main text are shown in figure S5 and summarised in table S2. The fitted parameters are shown in table S2.



This journal is © The Royal Society of Chemistry [year]

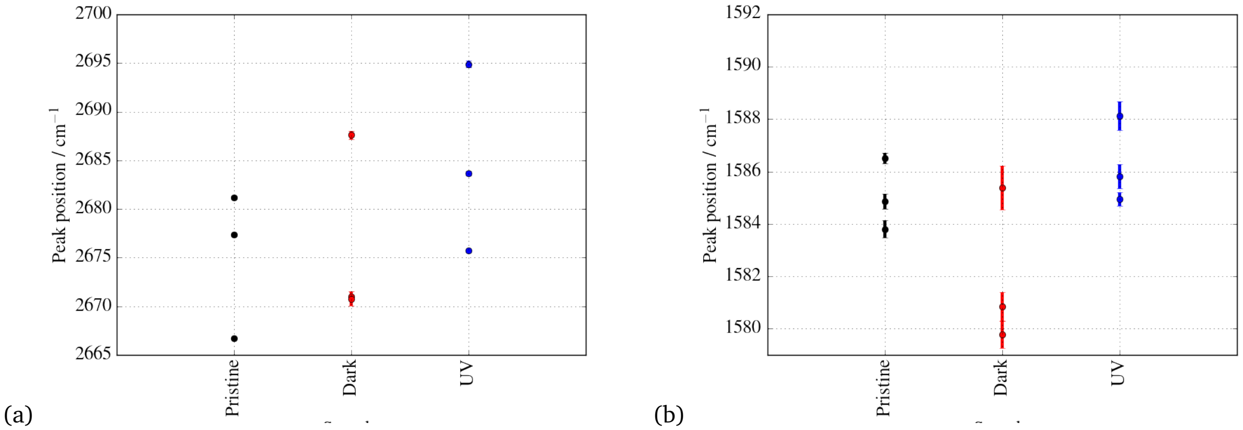


**Fig. S3** Showing up shift of (a) the G' peaks and (b) the G peaks for GFP Raman data.

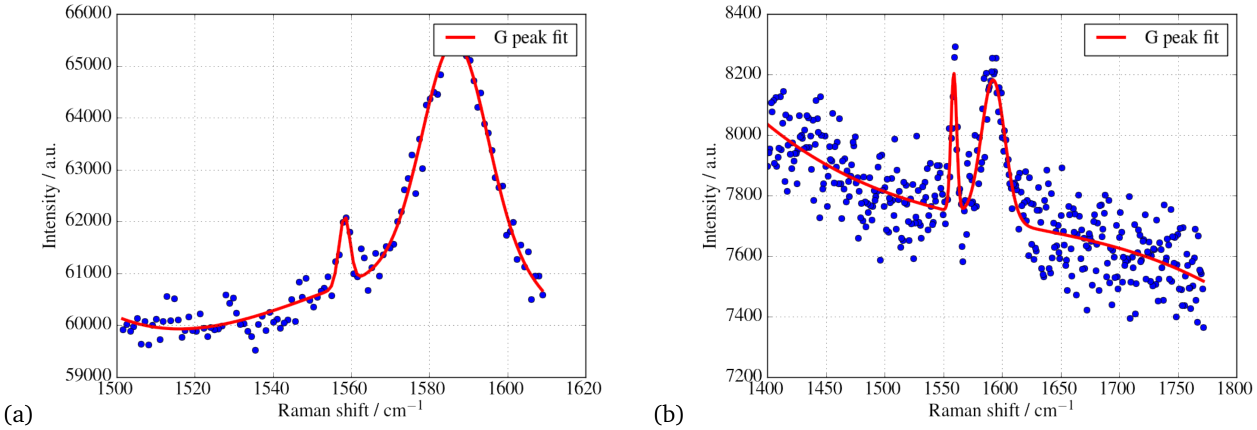


**Fig. S4** Details of G peak for UV-exposed samples. (a) A fit to raw GFP Raman data (blue, 40 accumulations of data) is shown as a red curve and comprises a double Gaussian peaks with a cubic polynomial background. A small peak is just visible at 1558.33±0.24 cm$^{-1}$. (b) Fit to cyt $b_{562}$ data.

| | G | | | G' | | |
|---|---|---|---|---|---|---|
| | Position / cm$^{-1}$ | Height (arb) | Width / cm$^{-1}$ | Position / cm$^{-1}$ | Height (arb) | Width / cm$^{-1}$ |
| **Pristine 1** | 1583.80 ± 0.33 | 1711.6 ± 39.9 | 12.64 ± 0.36 | 2666.71 ± 0.11 | 5369.7 ± 26.9 | 20.55 ± 0.13 |
| **Pristine 2** | 1586.50 ± 0.20 | 3073.0± 51.1 | 10.82 ± 0.22 | 2681.17 ± 0.13 | 5018.6 ± 30.4 | 19.41 ± 0.14 |
| **Pristine 3** | 1584.87 ± 0.28 | 2429.3 ± 55.9 | 10.69 ± 0.30 | 2677.36 ± 0.11 | 7064.3 ± 39.8 | 17.21 ± 0.12 |
| **Dark 1** | 1585.39 ± 0.38 | 1335.8 ± 38.6 | 11.71 ± 0.41 | 2682.34 ± 0.25 | 3824.7 ± 39.0 | 22.24 ± 0.28 |
| **Dark 2** | 1584.38 ± 0.29 | 3376.1 ± 67.4 | 12.75 ± 0.31 | 2675.49 ± 0.12 | 8602.1 ± 43.5 | 21.13 ± 0.13 |
| **Dark 3** | 1590.86 ± 0.86 | 405.1 ± 27.1 | 11.39 ± 0.93 | 2702.54 ± 0.54 | 706.6 ± 16.5 | 20.72 ± 0.59 |
| **Dark 5** | 1587.75 ± 0.27 | 1559.2 ± 43.9 | 8.49 ± 0.29 | 2683.56 ± 0.07 | 7756.1 ± 36.8 | 13.06 ± 0.07 |
| **UV 1** | 1588.29 ± 0.48 | 954.1 ± 35.4 | 11.54 ± 0.52 | 2685.60 ± 0.20 | 2409.8 ± 21.0 | 20.13 ± 0.21 |
| **UV 2** | 1589.81 ± 0.53 | 769.2 ± 29.4 | 12.39 ± 0.58 | 2697.10 ± 0.44 | 1032.7 ± 16.6 | 24.64 ± 0.49 |
| **UV 3** | 1589.16 ± 0.48 | 713.2 ± 30.1 | 10.02 ± 0.51 | 2688.86 ± 0.28 | 1580.3 ± 19.2 | 20.66 ± 0.31 |
| **UV 4** | 1592.69 ± 0.71 | 444.5 ± 30.5 | 9.08 ± 0.75 | 2710.37 ± 0.46 | 756.5 ± 16.1 | 18.89 ± 0.49 |

**Table S2** Comparison of main peak position, height and width for cyt $b_{562}$ Raman data shown in figure 11 of the main text. The fits are shown in figure S5.

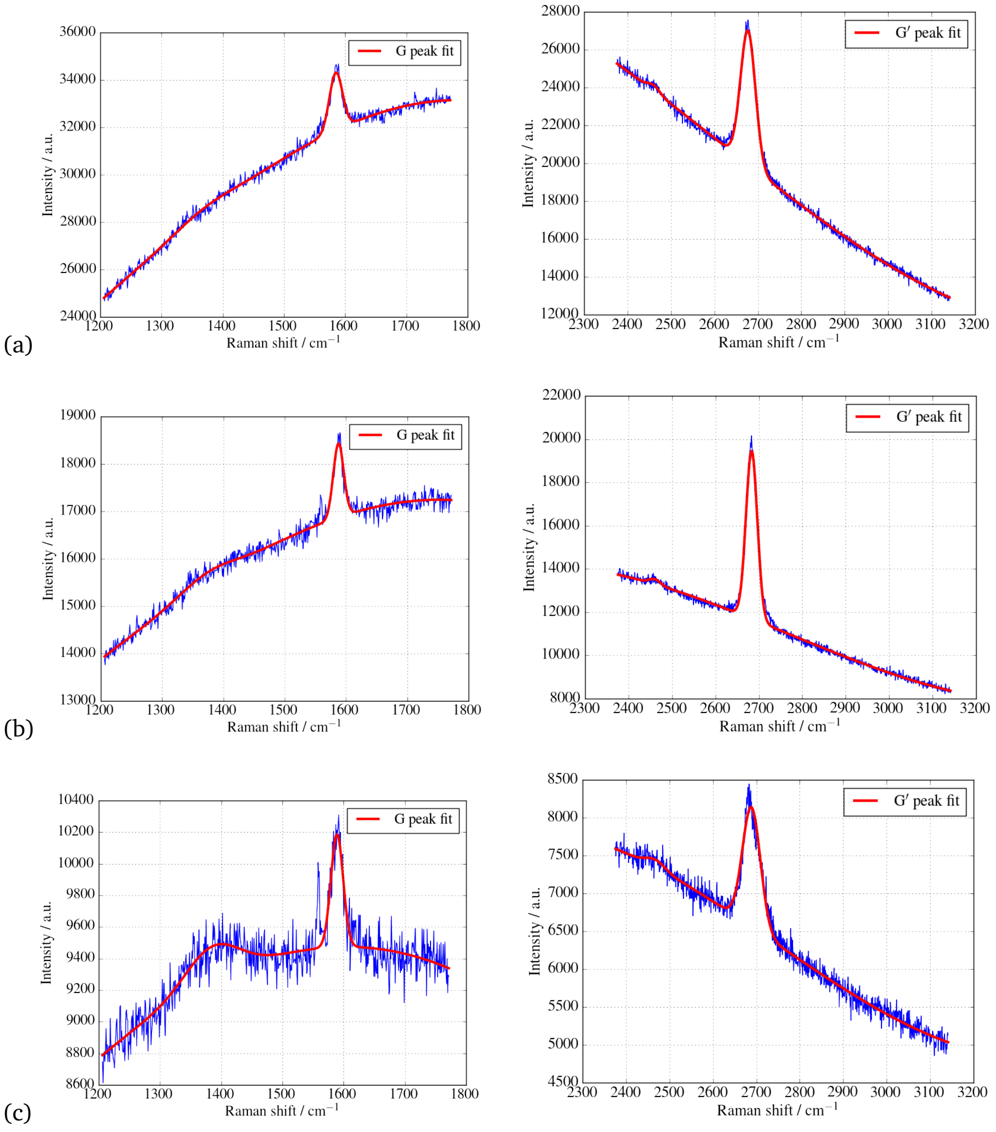


**Fig. S5** Selection of fits to raw Raman data (blue) are shown as red curves and comprise double Gaussian peaks with a cubic polynomial background. The left (or right) column shows data around the G' (or G) peaks. (a) pristine graphene (pristine 3) (b) with proteins deposited in the dark (dark 5) (c) with proteins deposited with UV exposure (UV 3). For clarity, the sharp feature below the G peak at around 1560 cm$^{-1}$ has not been fitted in these images.

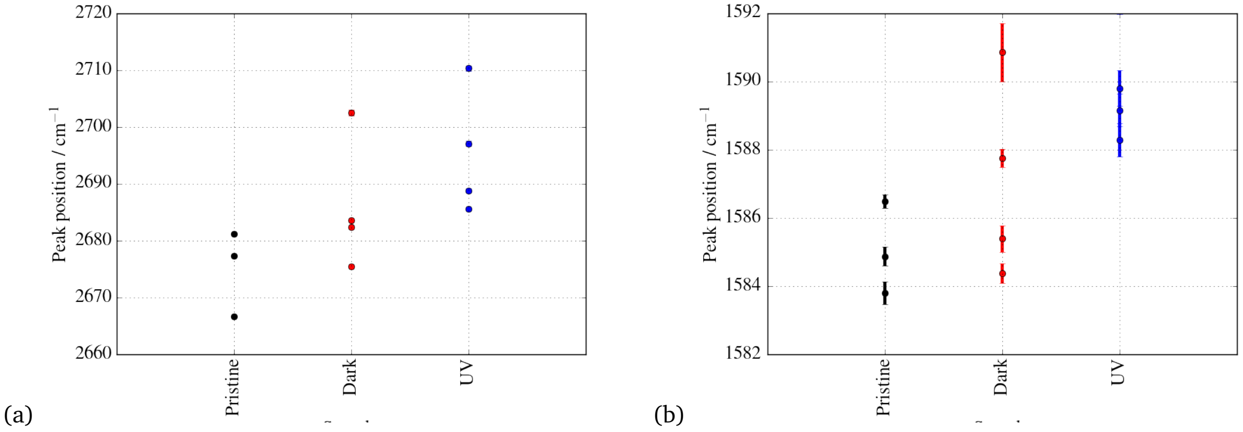


**Fig. S6** Showing up shift of (a) the G' peaks and (b) the G peaks for cyt $b_{562}$ Raman data.


This journal is © The Royal Society of Chemistry [year]
